# Integrating hormonal analyses into the development of hormonal male contraception: geographic centre as a determinant of individual response

**DOI:** 10.1093/hropen/hoag095

**Published:** 2026-10-23

**Authors:** Michael Zitzmann, Stefan Schlatt, Ebo Nieschlag, Ndema Habib, Mario Festin, David J Handelsman, Robert McLachlan, Maria Cristina Meriggiola, Gabriela Noé, Silvia Lestari, Hermann M Behre, Frederick Wu, Richard A Anderson

**Affiliations:** Centre for Reproductive Medicine and Andrology, University of Münster, Münster, Germany; Centre for Reproductive Medicine and Andrology, University of Münster, Münster, Germany; Centre for Reproductive Medicine and Andrology, University of Münster, Münster, Germany; Department of Sexual and Reproductive Health and Research, Maternal, Child, Adolescent Health and Ageing (Human Reproduction Program), World Health Organization, Geneva, Switzerland; Department of Sexual and Reproductive Health and Research, Maternal, Child, Adolescent Health and Ageing (Human Reproduction Program), World Health Organization, Geneva, Switzerland; Andrology Department, Concord Hospital, ANZAC Research Institute, University of Sydney, Sydney, NSW, Australia; Hudson Institute of Medical Research, Monash University, Melbourne, VIC, Australia; Gynecology and Physiopathology of Reproduction, IRCCS Azienda Ospedaliero Universitaria di Bologna, Bologna, Italy; Instituto Chileno de Medicina Reproductiva, Santiago, Chile; Faculty of Medicine, University of Indonesia, Jakarta, Indonesia; Centre for Reproductive Medicine and Andrology, University of Halle, Halle, Germany; Division of Endocrinology, Diabetes & Gastroenterology, University of Manchester, Manchester, UK; Centre for Reproductive Health, University of Edinburgh, Edinburgh, UK

**Keywords:** contraception, andrology, male contraception, testosterone, adverse events

## Abstract

**STUDY QUESTION:**

Are the geographic recruitment centre and dynamic sex-steroid profiles, particularly for dihydrotestosterone, associated with inter-individual differences in libido and mood-related adverse events during hormonal male contraception?

**SUMMARY ANSWER:**

In this multinational analysis of the WHO testosterone undecanoate/norethisterone (TU/NETE) enanthate trial, geographic cluster was associated with distinct androgenic profiles and clinical responses; lower dihydrotestosterone exposure emerged as a consistent predictor of decreased libido and mood-related adverse events.

**WHAT IS KNOWN ALREADY:**

Combined testosterone–progestin regimens can suppress spermatogenesis effectively, but the WHO TU/NETE trial was discontinued prematurely because of mood-related adverse events despite high contraceptive efficacy. The endocrine mechanisms underlying these adverse effects remain incompletely understood.

**STUDY DESIGN, SIZE, DURATION:**

This secondary analysis used serial hormone and adverse-event data from the multinational WHO/CONRAD trial, which enrolled 320 men. However, 255 participants had serum samples available for centralized steroid analysis and constituted the present analytical cohort, as the samples from Jakarta, Indonesia (65 subjects) were not sent to the Central Laboratory.

**PARTICIPANTS/MATERIALS, SETTING, METHODS:**

Healthy male participants were recruited across European/Australian, South American, and Asian centres and grouped into geographic clusters. Serum testosterone, calculated free testosterone, dihydrotestosterone, estradiol, and norethisterone were analysed centrally by gas chromatography–mass spectrometry (GC-MS). Longitudinal mixed-effects models and time-to-event analyses were used to evaluate associations between hormone trajectories, geographic cluster, age, libido changes, and mood disorders.

**MAIN RESULTS AND THE ROLE OF CHANCE:**

Marked cluster-dependent heterogeneity was observed. Participants from Chile showed higher total and free testosterone and persistently higher dihydrotestosterone concentrations over time, accompanied by a higher incidence of increased libido. Participants from India showed lower dihydrotestosterone exposure and higher norethisterone concentrations and no libido and mood changes. European/Australian participants showed comparatively lower dihydrotestosterone concentrations and the highest cumulative incidence of mood disorders. Declining dihydrotestosterone concentrations were associated with decreased libido and mood-related adverse events.

**LIMITATIONS, REASONS FOR CAUTION:**

The analyses are exploratory and based on secondary evaluation of a completed clinical trial. Geographic clusters may reflect genetic, environmental, dietary, cultural, behavioural, and reporting-related factors, and causality cannot be inferred.

**WIDER IMPLICATIONS OF THE FINDINGS:**

Comprehensive androgen profiling, particularly assessment of dihydrotestosterone, may help identify men at increased risk of neuropsychological or sexual adverse effects during hormonal male contraception and may support more individualized contraceptive regimens.

**FUNDING:**

The clinical study was cosponsored and funded by United Nations Development Programme; United Nations Population Fund; United Nations International Children’s Emergency Fund; World Health Organization; World Bank Special Programme of Research, Development, and Research Training in Human Reproduction (Human Reproduction Programme, World Health Organization, Geneva, Switzerland); and CONRAD (Eastern Virginia Medical School, Arlington, VA using funding from the Bill & Melinda Gates Foundation and U.S. Agency for International Development).

**DISCLOSURES:**

R.I.M.is a Principal Research Fellow of the Australian National Health and Medical Research Council (1022327) and the Hudson Institute, which is supported by the Victorian Government’s Operational Infrastructure Scheme. The authors have no conflicts of interest to disclose.

**TRIAL REGISTRATION NUMBER:**

N/A.

WHAT DOES THIS MEAN FOR PATIENTS? Hormonal contraception for men is still experimental and is not currently available as a routine method of birth control. This study helps us understand why men may respond differently to such treatments.

In a previous international trial, injections containing testosterone and a progestogen were highly effective at suppressing sperm production, but the study was stopped early because some participants experienced changes in mood, emotional well-being or sexual desire.

By looking more closely at hormone levels during treatment in that previous trial, the present study suggests that it may be the way an individual man’s hormones change over time, rather than his hormone levels before treatment, that is particularly important. Changes in dihydrotestosterone, a hormone made from testosterone, were associated with changes in libido and with mood-related side effects, although the study cannot prove that these hormonal changes were their direct cause.

Men from different study centres also responded differently, but these differences should not be interpreted simply as effects of ethnicity or geography, since lifestyle, diet, environment, culture, and other factors may all contribute.

For patients and couples, the encouraging message is that future male contraceptives may not need to follow a ‘one size fits all’ approach: monitoring how hormones and symptoms change during treatment could eventually help researchers design contraceptive methods that are not only effective, but also better tailored to the individual man and therefore more acceptable and safer to use.

## Introduction

The development of effective and safe hormonal male contraceptive options has become an increasingly prominent focus in reproductive health research. This paper examines the challenges and adverse effects that arose during a World Health Organization (WHO) trial on hormonal male contraception (Behre *et al.*, 2016), which was prematurely discontinued despite demonstrating contraceptive efficacy. The WHO trial represented a landmark advance in hormonal male contraceptive research, confirming high efficacy in pregnancy prevention; however, its termination on safety grounds, primarily changes in libido and mood, underscores the complexity of hormonal regulation in men and raises critical questions regarding the acceptability of adverse effects in male contraception.

Early clinical trials investigating testosterone enanthate (TE) as a sole agent for male hormonal contraception established that exogenous testosterone could suppress gonadotropin secretion sufficiently to impair spermatogenesis. Weekly intramuscular administration of TE achieved azoospermia in ∼75% of participants, with the remainder reaching severe oligozoospermia (WHO Task Force on Methods for the Regulation of Male Fertility, 1990, 1996). These findings confirmed the biological principle that sustained supraphysiological circulating testosterone concentrations exert negative feedback on the hypothalamic–pituitary–gonadal axis, thereby suppressing endogenous testicular testosterone production and disrupting spermatogenesis. The incomplete azoospermic response in a significant minority highlighted the need for more potent suppression strategies, motivating development of combined testosterone–progestogen regimens (Anderson and Baird, 2002; Anderson and Reynolds-Wright, 2024).

Although weekly intramuscular TE can induce effective and reversible azoospermia in most men, the dose required frequently exceeds physiological testosterone production and may be associated with metabolic adverse effects. The addition of a depot progestin markedly increased the proportion achieving complete suppression (90% azoospermic, 100% severely oligozoospermic) without additional androgenic or metabolic adverse effects (Handelsman *et al.*, 1996). Progestins act synergistically with testosterone by further suppressing LH and FSH secretion, thereby enhancing inhibition of intratesticular testosterone production and spermatogenesis. These findings established the rationale for combined androgen–progestin regimens (Turner *et al.*, 2003).

Liu *et al.* (2008) provided an integrated analysis of spermatogenic suppression during hormonal male contraception. Subsequent investigations then extended this evidence base: Amory *et al.* (2023) described a self-administered daily transdermal gel combining testosterone and segesterone acetate (Nestorone); Anawalt *et al.* (2019) characterized the efficacy of the nestorone–testosterone gel in suppressing serum gonadotropins; Mommers *et al.* (2008) contributed a double-blind, placebo-controlled trial providing a rigorous comparative framework for evaluating adverse-event profiles; Nguyen *et al.* (2020, 2021) explored acceptability of oral prototypes; Zitzmann *et al.* (2017; Zitzmann, 2018) investigated various progestins and cardiovascular risks; and Aaltonen *et al.* (2007) and Liu *et al.* (2006) provided regulatory recommendations and characterized spermatogenic recovery.

The WHO trial enrolled 320 healthy, fertile men aged 18–45 years at multiple international centres. Participants received intramuscular injections of 200 mg norethisterone enanthate (NET-EN; 200 mg/ml) and 1000 mg testosterone undecanoate (TU; 250 mg/ml) every 8 weeks during the suppression phase (up to 24 weeks) and, among participants entering efficacy, during the efficacy phase (up to 56 weeks) for a maximum combined injection period of ∼80 weeks. The primary endpoint was suppression of sperm concentration to below 1 million/ml (the threshold regarded as contraceptive) (Aaltonen *et al.*, 2007; Wang *et al.*, 2024), with contraceptive efficacy in the participating couples as a co-primary outcome. At the end of the 24-week suppression phase, 89.2% of participants had achieved a sperm concentration below 1 million/ml and were eligible to enter the efficacy phase.

Despite a generally favourable overall safety profile, the decision to halt the study was driven primarily by a subset of participants who experienced severe emotional disturbances, mood swings, and depression, some of whom required psychiatric intervention. The specific mechanisms underlying these emotional adverse effects remain unclear and warrant further investigation. This manuscript therefore examines the reported adverse effects in relation to serum sex steroid concentrations measured by GC-MS in the heterogeneous multinational cohort, with additional consideration of age and geographic recruitment centre as modifying factors.

## Materials and methods

### Study population and geographic clusters

The composition of the trial population by country of recruitment, together with full details of ethical approval and informed consent procedures, has been described previously (Behre *et al.*, 2016). Participants from Germany, Italy, and the UK constituted a substantial proportion of the study population, with additional men recruited from Australia, India, and Chile. For all analyses, participants are grouped into three geographic clusters: Cluster A: European and Australian centres (Germany, Italy, the UK, Australia) with 151 subjects; Cluster B: South American centre (Chile) with 63 subjects; and Cluster C: Asian centre (India) with 41 subjects.

This study is a secondary analysis of the multinational WHO/CONRAD trial reported by Behre *et al.* The parent trial enrolled 320 men. The present analysis is restricted to the 255 participants for whom serial serum samples suitable for centralized steroid analysis were available, as blood samples of the 65 subjects from Jakarta, Indonesia were not sent to the central lab and were not analysed. The parent trial population and the analytical population are therefore related but not numerically identical.

These groups include geographically, culturally, and ecologically distinct settings so that between-group differences in hormonal profiles and clinical outcomes may reflect genetic, environmental, nutritional, lifestyle, and cultural factors inherent to the respective recruitment regions. An age stratification was performed *a priori* as being clinically plausible, separating very young men (≤25 years, 43 subjects), men in early mature adulthood (>25 to ≤35 years, 133 subjects), and older participants (>35 years, 79 subjects). The overall median age was 32 years (range 18–45 years, interquartile range [IQR] was 27–36 years); age distribution across clusters was similar; and no significant difference existed. The median age and IQRs were: Cluster A: 33 years, IQR 26–39 years; Cluster B: 31 years, IQR 28–35 years; Cluster C: 30 years, IQR 25–36 years. Cluster A was specified *a priori* as the reference category because it comprised the largest analytical group and therefore provided the most stable comparator for the smaller single-country clusters. Selection as the reference was a modelling convention and should not be interpreted as designating Cluster A as biologically or clinically normative.

### Laboratory analyses

GC-MS was employed for sex-steroid analysis in participant serum owing to its high sensitivity, specificity, and broad analyte range. All analyses were performed centrally in Münster, Germany; samples were shipped on dry ice with an uninterrupted cold chain (Kannenberg *et al.*, 2018). The analyte panel comprised testosterone, estradiol, dihydrotestosterone (DHT), and the synthetic norethisterone. Concentrations of ethinylestradiol, a putative metabolite of norethisterone, were also measured. Free testosterone (FT) was calculated from total testosterone and SHBG and fixed albumin at 43 g/l using the Vermeulen equation (Vermeulen *et al.*, 1999). The term cFT is used throughout to distinguish the calculated estimate from directly measured free testosterone. Figure 1 presents analytic sensitivity and precision data. Serum LH, FSH, SHBG, and PSA were determined by chemiluminescent microparticle immunoassay (Architect i1000, Abbott Diagnostics, Wiesbaden, Germany) and are reported in Behre *et al.* (2016).

**Figure 1.**
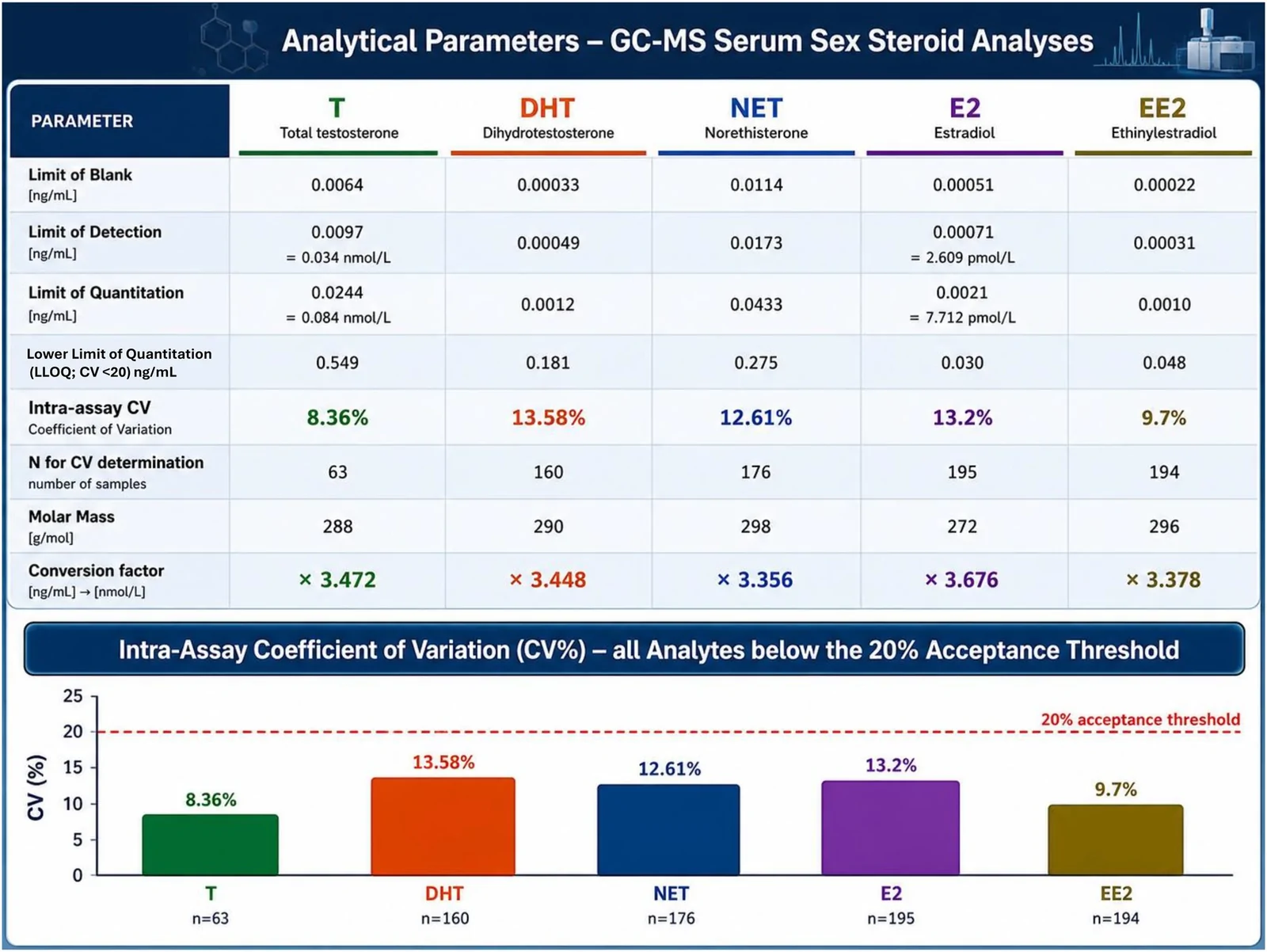
**Analytical performance data**. Analytical performance data for serum sex steroid quantification by gas chromatography–mass spectrometry (GC-MS) at the central laboratory in Münster, Germany. The table presents limits of blank (LoB), detection (LoD), and quantitation (LoQ) in ng/ml with molar conversions, the intra-assay coefficient of variation (CV%), sample counts (N) used for CV determination, molar masses (g/mol), and mass-to-molar conversion factors for five analytes: Testosterone (T), Dihydrotestosterone (DHT), Norethisterone (NET), Estradiol (E2), and Ethinylestradiol (EE2). The bar chart confirms that all intra-assay CVs are below the pre-specified 20% acceptance threshold. EE2 was quantifiable in only 13 samples of 11 participants. These people did not report adverse events. This figure reports the limit of blank (LoB), limit of detection (LoD), and limit of quantitation (LoQ) as distinct analytical characteristics. The additional label denotes the lower limit of quantitation at which the prespecified precision criterion of CV <20% was fulfilled; it does not indicate that LoD and LoQ are equivalent.

### Statistical approaches

Statistical analyses were conducted using SAS/STAT v9.4 (SAS Institute Inc., SAS Campus Drive, Cary, NC, USA); survival graphics and box-and-whisker plots were generated in R (The R Foundation for Statistical Computing, Vienna, Austria). Box-and-whisker plots were constructed from enrolment to the last study visit, grouped by visit type within the suppression, efficacy, and recovery phases, for: calculated free testosterone (cFT, pmol/l), total testosterone (nmol/l), DHT (ng/l), norethisterone (ng/l), and estradiol (ng/ml). Cluster-by-time interaction plots from the date of first injection were displayed for each hormone. SAS PROC MIXED was used to fit multilevel regression models incorporating random intercepts and slopes to assess statistical significance at the 5% level, with error variance reported for all models.

For time-to-event outcomes, cumulative hazard rate probabilities were computed using the Kaplan–Meier product-limit method (Lee, 1992). Both fixed- and time-varying proportional hazards models were applied to assess effects of hormone concentrations on the rate of first incidence of increased libido, decreased libido, and mood disorders. Point estimates and 95% confidence limits are reported throughout; results were considered statistically significant at *P* < 0.05. *Post hoc* comparisons employed Games–Howell tests; Levene’s test, and the Breusch–Pagan test assessed variance homogeneity and heteroscedasticity, respectively.

## Results

The adverse events accumulated during the trial and used in the present analysis are shown in Fig. 2. Figure 3 presents serum concentrations across the full cohort throughout the trial period for total testosterone and norethisterone (the administered study drugs). Figure 4 exhibits serum concentrations of sex steroid metabolites (dihydrotestosterone, estradiol, and cFT). Ethinylestradiol (EE2) was quantifiable in 13 samples from 11 participants; two participants had quantifiable concentrations at two time points. These men did not report any adverse events.

**Figure 2.**
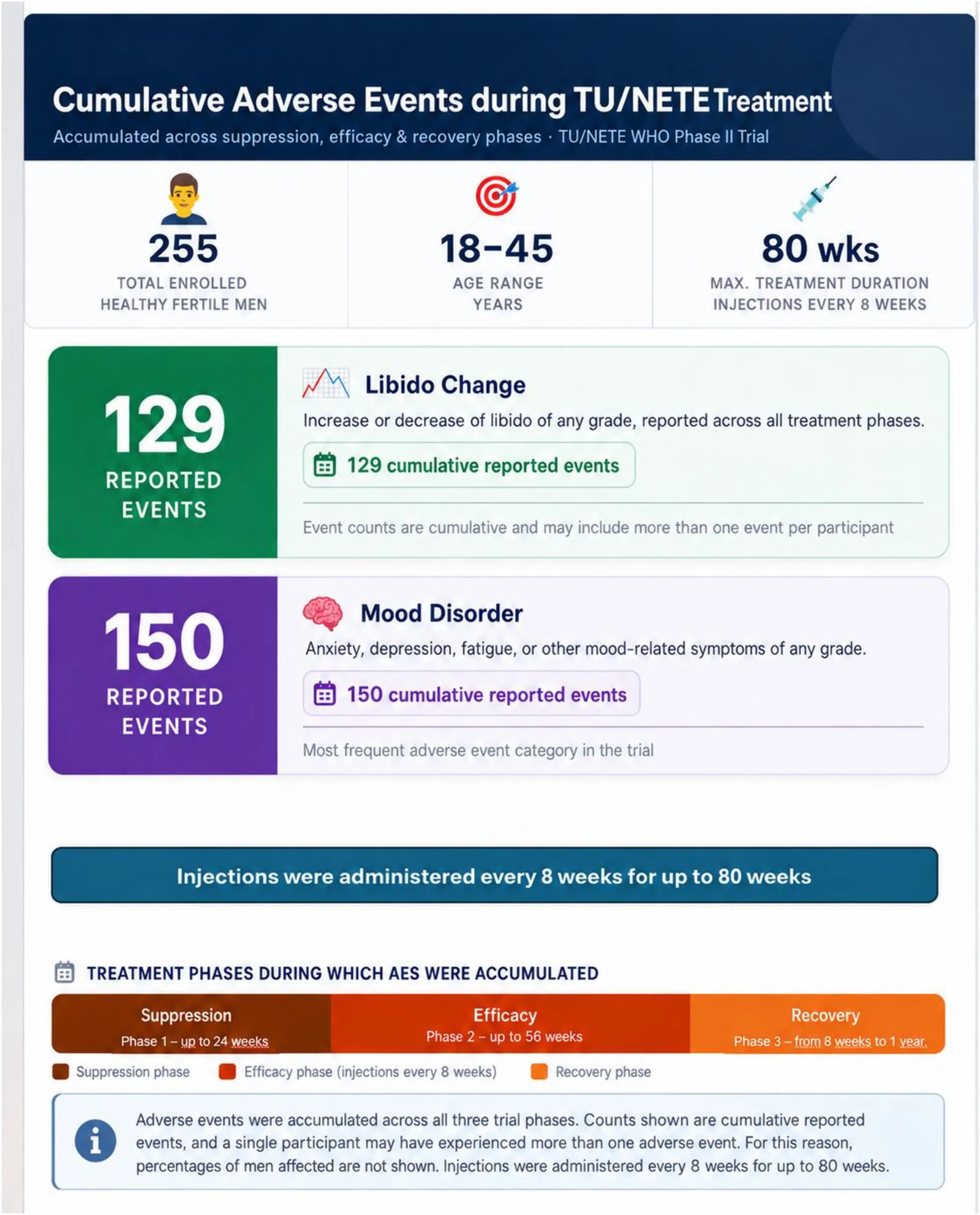
**Principal adverse event categories**. Overview of the two principal adverse event categories, libido change and mood disorder, accumulated across all three trial phases (Suppression, Efficacy, Recovery) in 255 enrolled healthy fertile men aged 18–45 years. Injections were administered every 8 weeks for up to 80 weeks (up to 24 weeks for suppression plus up to 56 weeks for efficacy). Libido change (increase or decrease) affected 129/255 participants (50.5%); mood disorder (anxiety, depression, fatigue, or other mood-related symptoms of any grade) affected 150/255 participants (58.8%). Events are cumulative first-onset and forward counts; individual participants may have been counted in more than one category. The analytical cohort comprised 255 men: Cluster A, n = 151; Cluster B, n = 63; and Cluster C, n = 41. Increased libido was recorded 54 times in 46 participants in Cluster A (30% of subjects affected), 72 times in 47 participants in Cluster B (65% of subjects affected), and zero times in Cluster C. Decreased libido was recorded 13 times in 12 participants in Cluster A (8% of subjects affected) and zero times in Clusters B and C. Possible limitation: The study was not prospectively powered for comparisons of adverse-event rates among the three geographic clusters while the method of analysis was designed to provide a respective power of 90% to detect changes at a level of *P* = 0.05. Cluster-level estimates should therefore be interpreted as exploratory signals rather than precise population-specific effects.

**Figure 3.**
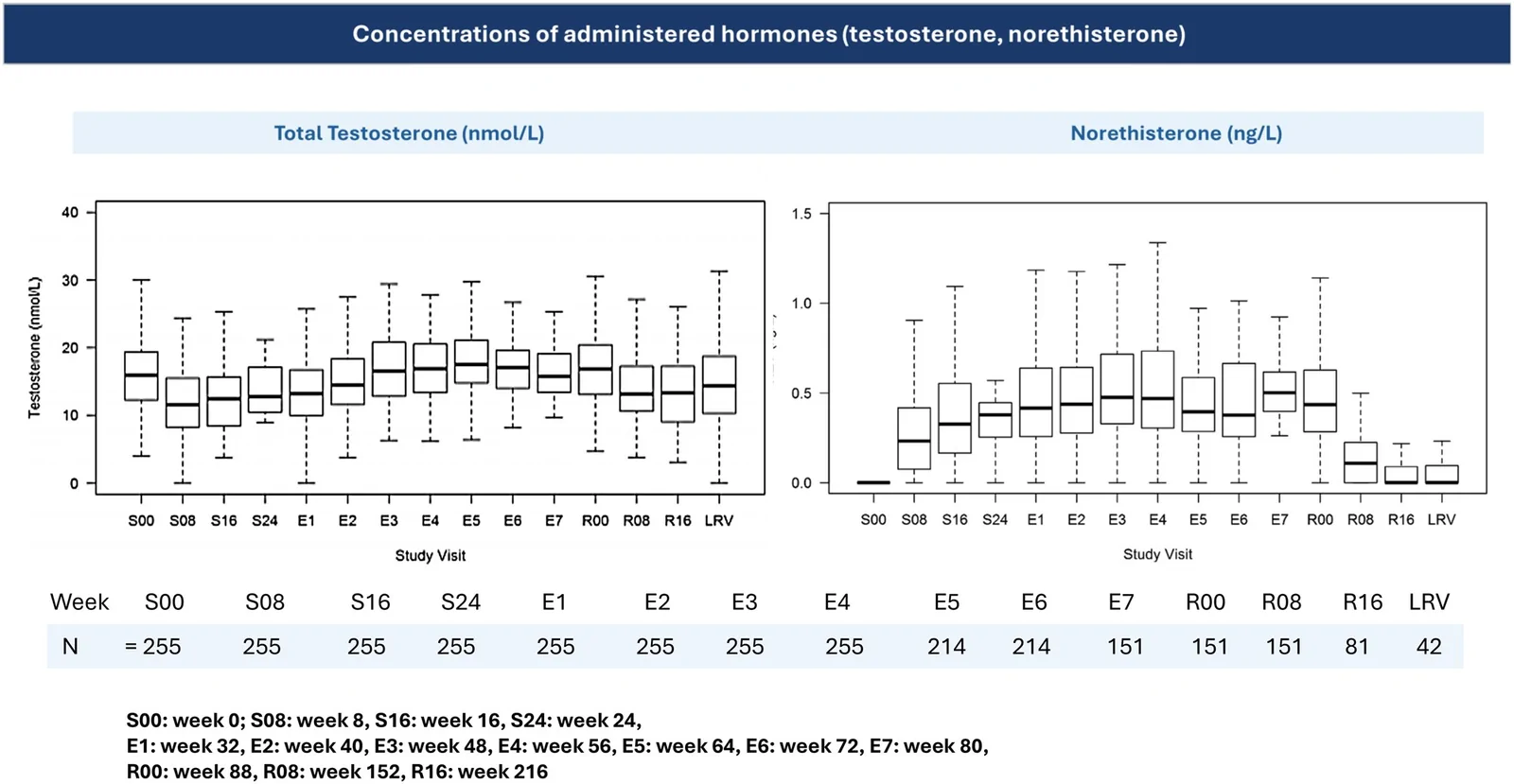
**Concentrations of administered hormones**. Box-and-whisker plots showing concentrations of administered hormones across study visits. Total testosterone is displayed in nmol/l and norethisterone in ng/l. Visit labels S00–S24 denote suppression-phase visits, E1–E7, efficacy-phase visits, R00–R16, recovery-phase visits, and LRV, the late recovery/final recovery visit.

**Figure 4.**
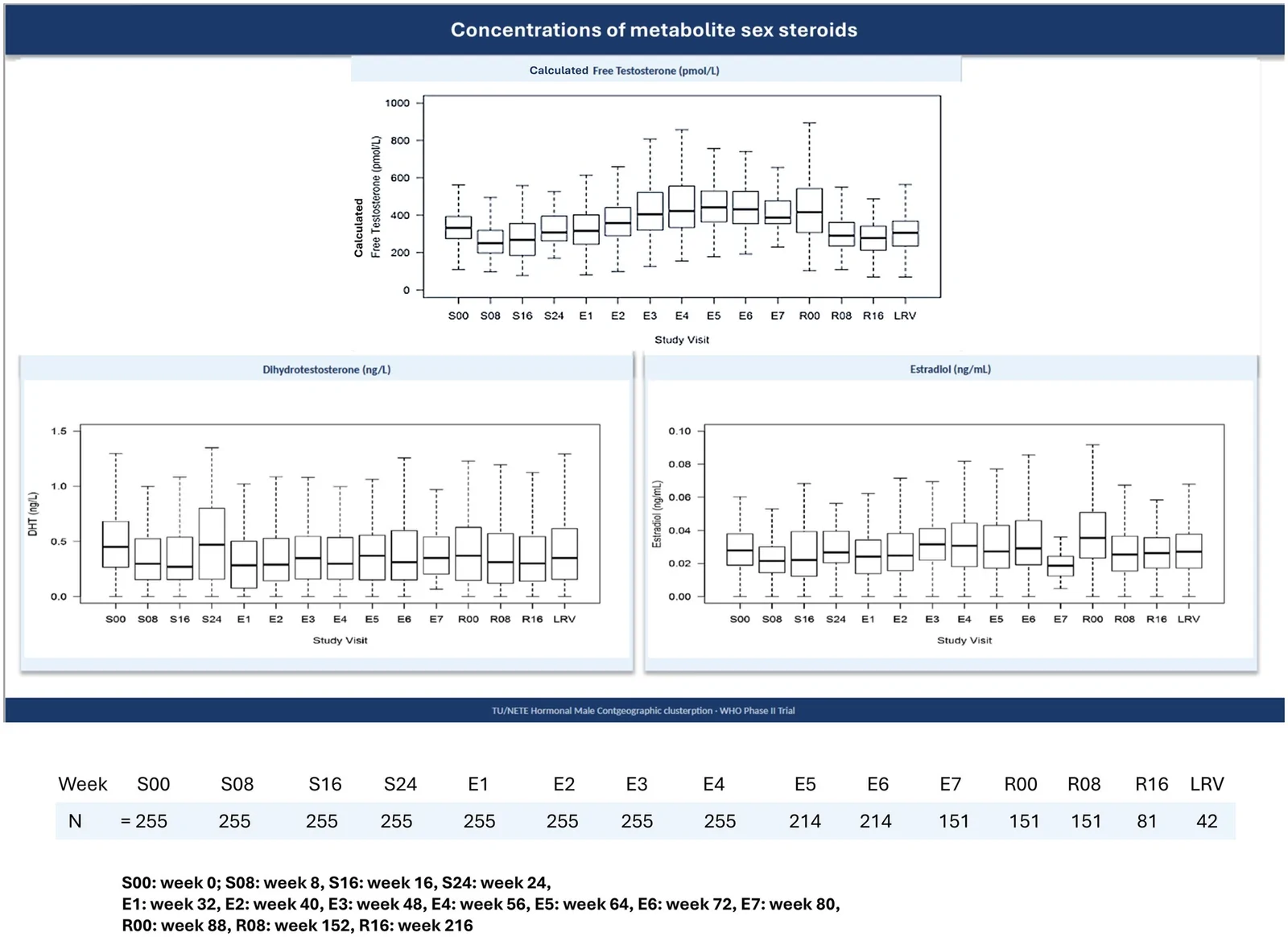
**Concentrations of hormone metabolites**. Box-and-whisker plots showing concentrations of metabolite sex steroids across study visits. Free testosterone as calculated according to Vermeulen (cFT) is shown in pmol/l, dihydrotestosterone (DHT) in ng/l, and estradiol (E2) in ng/ml. Visit labels S00–S24 denote suppression-phase visits, E1–E7, efficacy-phase visits, R00–R16, recovery-phase visits, and LRV, the late recovery/final recovery visit.

The GC-MS findings in relation to participant complaints, and their associations with age and geographic cluster are outlined below, highlighting the statistically significant outcomes (all other evaluated associations were non-significant):

Both total and calculated free testosterone concentrations were lower in Cluster A participants than in Cluster B (Figs 5 and 6). In addition, DHT concentrations were higher in Cluster B than in Cluster A, and lower in Cluster C than Cluster A (Fig. 7). Figure 8 demonstrates that serum norethisterone concentrations differed by geographic cluster; concentrations in Cluster C were higher than in Clusters A and B.

**Figure 5.**
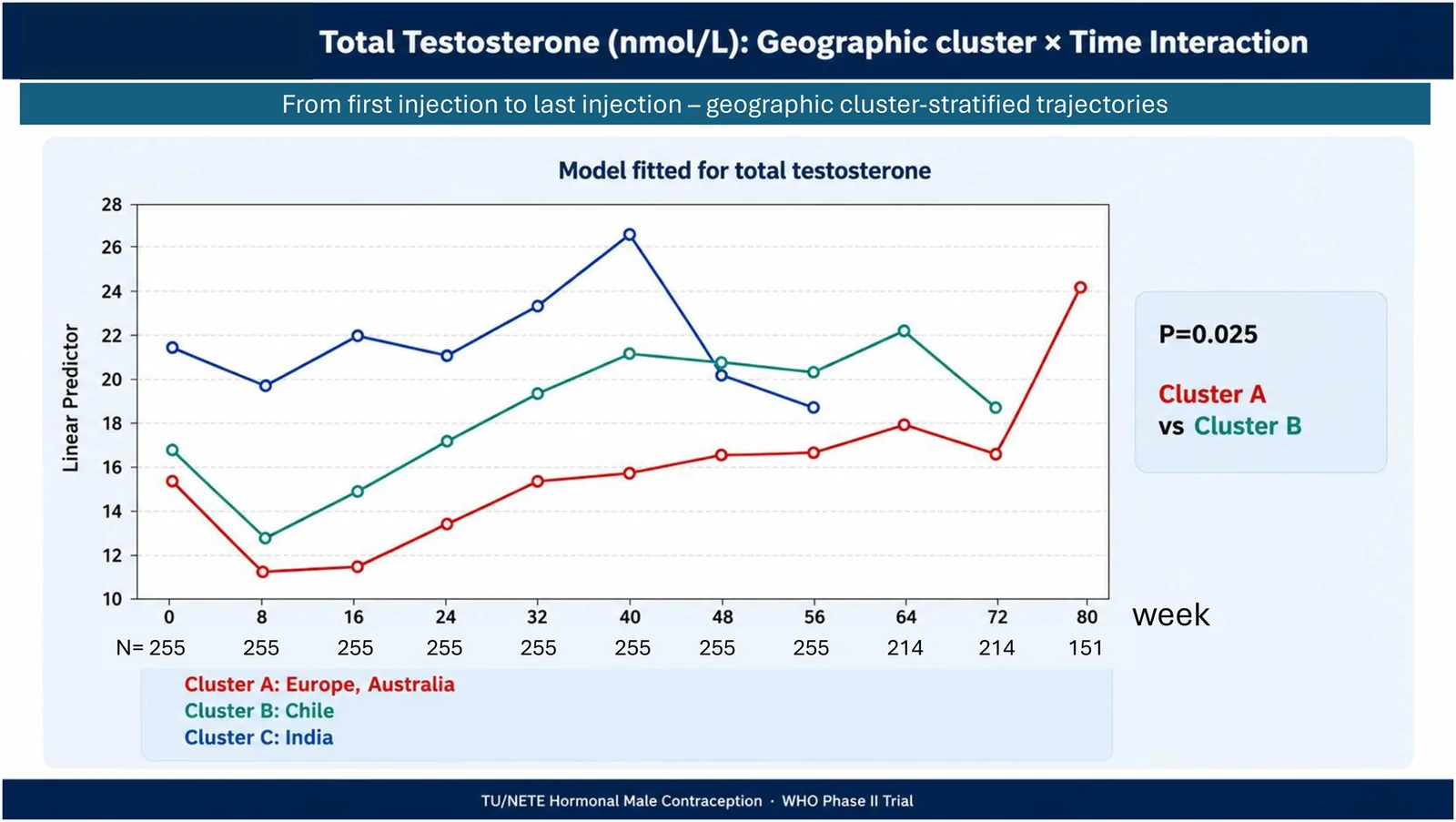
**Mixed-effects model-fitted trajectories for total testosterone**. Mixed-effects model-fitted trajectories for total testosterone (TT) from first injection to 8 weeks after the last injection, stratified by geographic cluster. Cluster A comprised Europe and Australia, Cluster B, Chile, and Cluster C, India. The highlighted comparison indicates a significant difference between Cluster A and Cluster B (*P* = 0.025). The plotted series were constructed from analyte-specific stored-sample availability, and different analytes do not necessarily share identical denominators at a given visit. The values not shown are due to missing samples. Cluster C (India) stopped sampling at week 56. Reported *P*-values distinguish overall cluster effects from cluster-by-time interactions. They should not be interpreted as evidence that pairwise differences were statistically significant at every visit unless explicitly stated.

**Figure 6.**
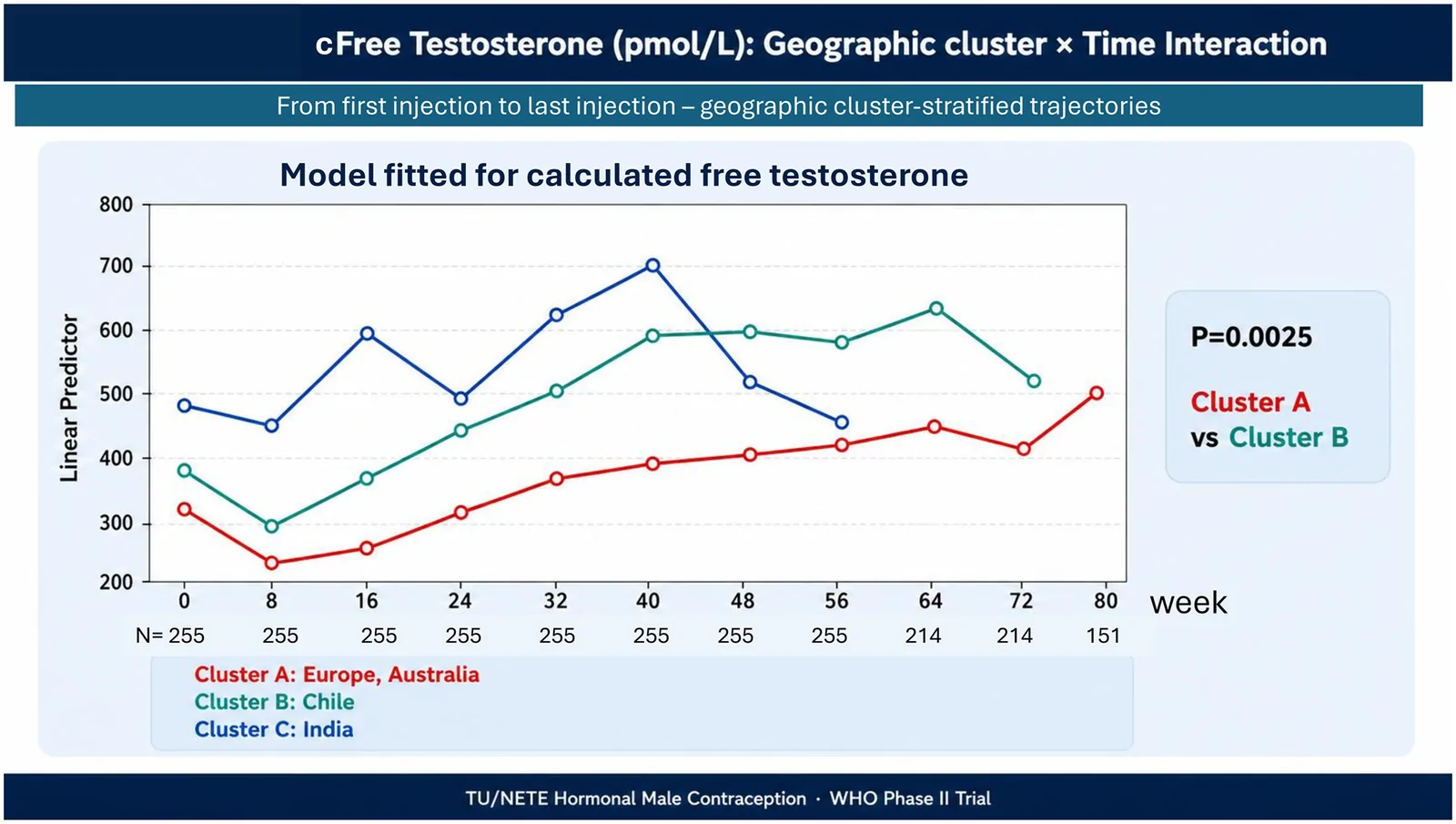
**Mixed-effects model-fitted trajectories for calculated free testosterone**. Mixed-effects model-fitted trajectories for calculated free testosterone (cFT; calculated according to Vermeulen) by geographic cluster over time. Cluster definitions were as follows: Cluster A, Europe/Australia; Cluster B, Chile; Cluster C, India. A significant difference between Cluster A and Cluster B is indicated (*P* = 0.0025). The plotted series were constructed from analyte-specific stored-sample availability, and different analytes do not necessarily share identical denominators at a given visit. The values not shown are due to missing samples. Cluster C (India) stopped sampling at week 56. Reported *P*-values distinguish overall cluster effects from cluster-by-time interactions. They should not be interpreted as evidence that pairwise differences were statistically significant at every visit unless explicitly stated.

**Figure 7.**
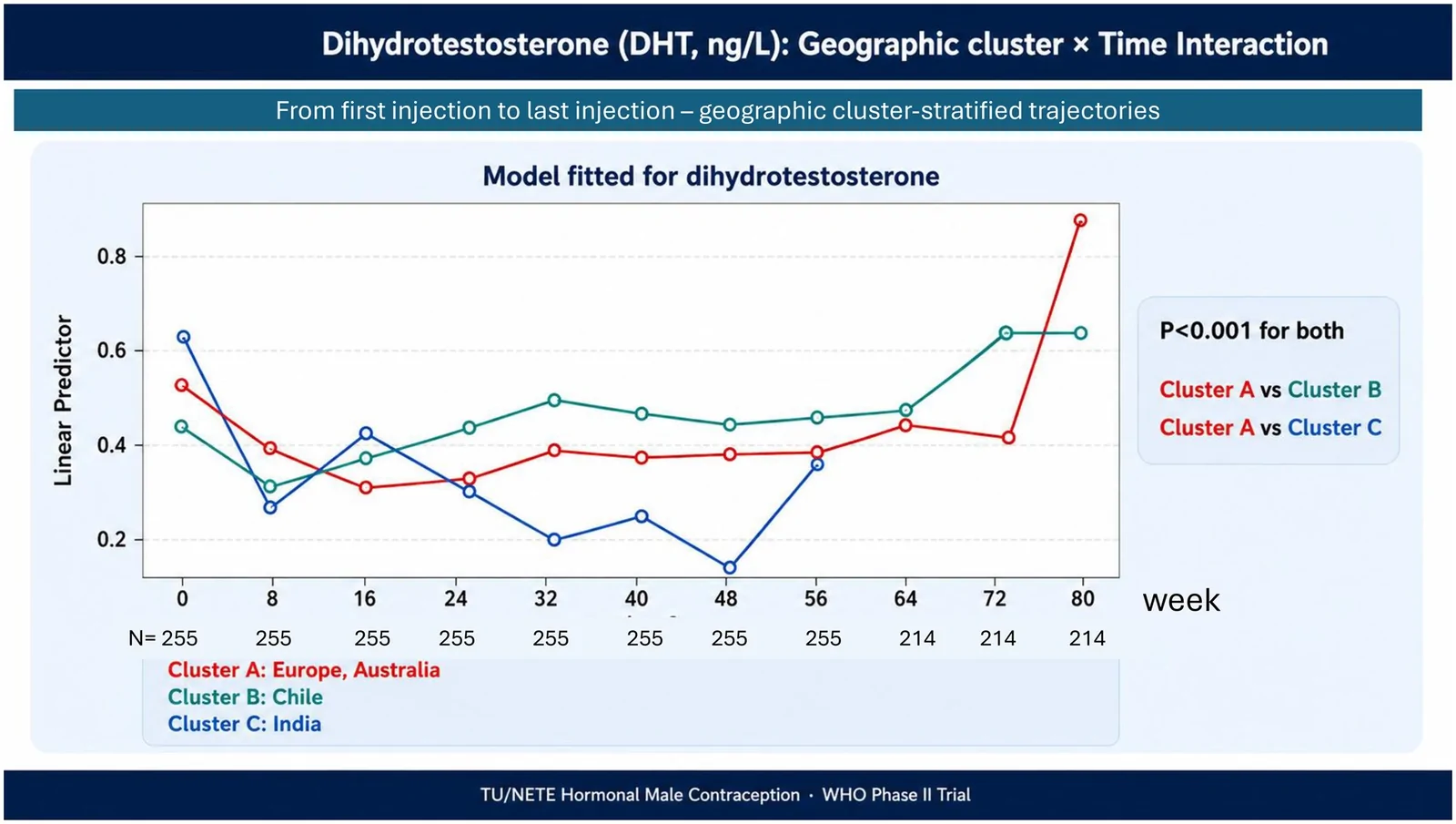
**Mixed-effects model-fitted trajectories for dihydrotestosterone**. Mixed-effects model-fitted trajectories for dihydrotestosterone (DHT) by geographic cluster over time. Cluster definitions were as follows: Cluster A, Europe/Australia; Cluster B, Chile; Cluster C, India. Significant differences were observed for Cluster A versus Cluster B and for Cluster A versus Cluster C (both *P* < 0.001). The plotted series were constructed from analyte-specific stored-sample availability, and different analytes do not necessarily share identical denominators at a given visit. The values not shown are due to missing samples. Cluster C (India) stopped sampling at week 56. Reported *P*-values distinguish overall cluster effects from cluster-by-time interactions. They should not be interpreted as evidence that pairwise differences were statistically significant at every visit unless explicitly stated.

**Figure 8.**
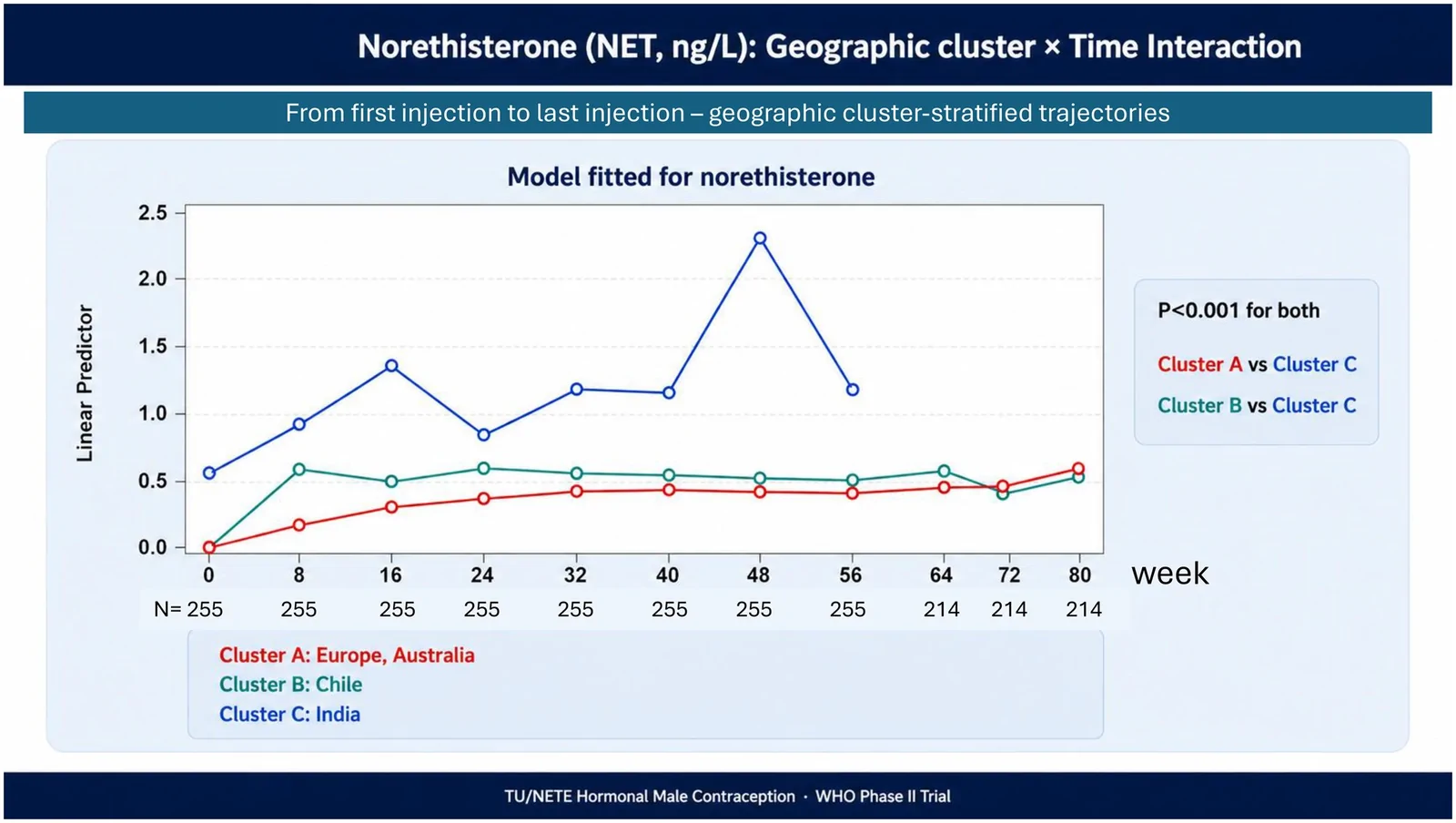
**Mixed-effects model-fitted trajectories for norethisterone**. Mixed-effects model-fitted trajectories for norethisterone (NET) by geographic cluster over time. Cluster definitions were as follows: Cluster A, Europe/Australia; Cluster B, Chile; Cluster C, India. A significant difference was observed between Cluster A and Cluster C as well as between Cluster B and Cluster C (*P* < 0.001). The plotted series were constructed from analyte-specific stored-sample availability, and different analytes do not necessarily share identical denominators at a given visit. The values not shown are due to missing samples. Cluster C (India) stopped sampling at week 56. Reported *P*-values distinguish overall cluster effects from cluster-by-time interactions. They should not be interpreted as evidence that pairwise differences were statistically significant at every visit unless explicitly stated.

Corroborative, multilevel mixed regression models demonstrated significantly higher serum levels for total and free testosterone in Clusters B and C compared with Cluster A (Fig. 9). This was also seen for location-time-interaction regarding Cluster B versus Cluster A (Fig. 9).

**Figure 9.**
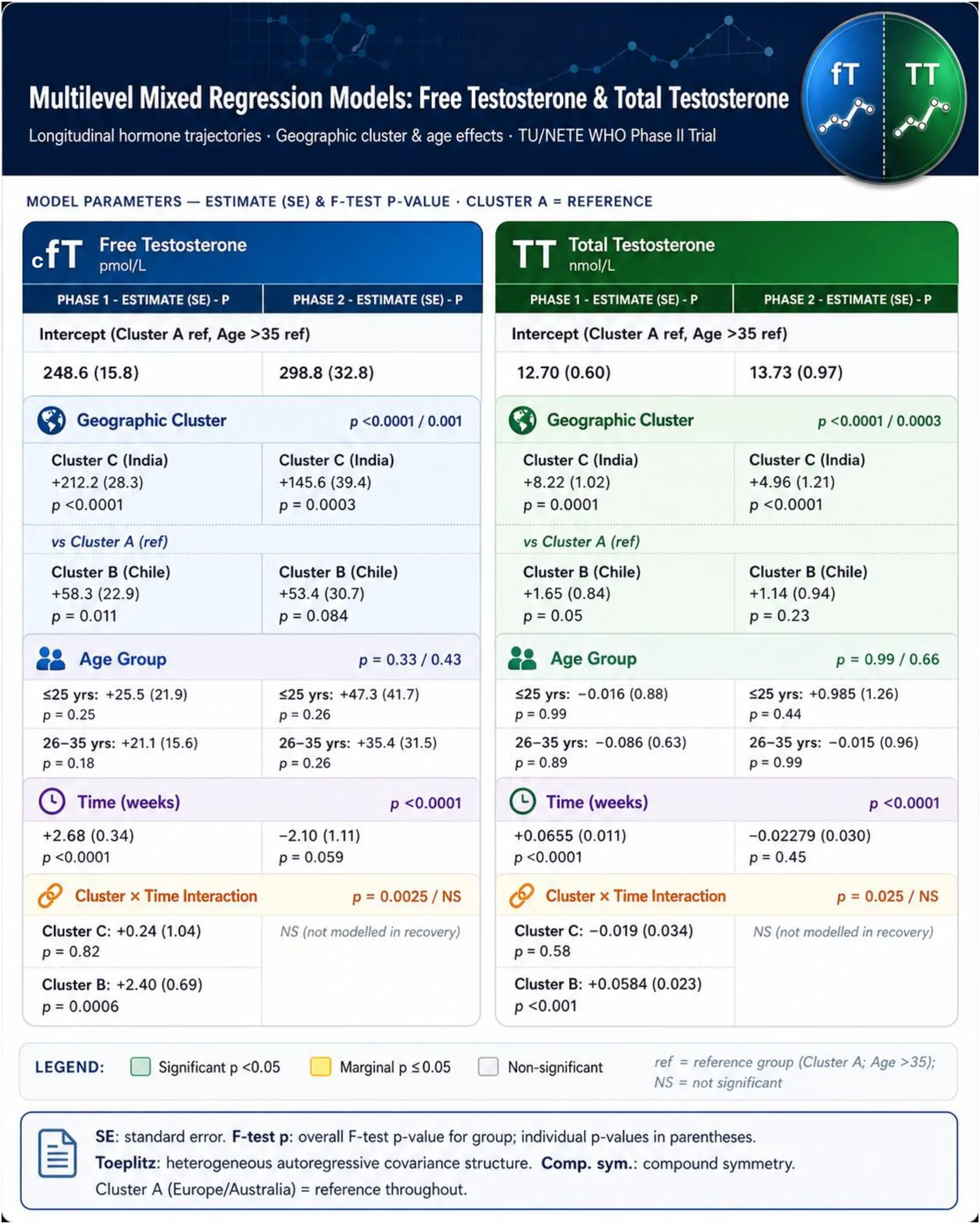
**Multilevel mixed-effects regression models for calculated serum free testosterone and total testosterone**. Results of multilevel mixed-effects regression models for calculated serum free testosterone (cFT, pmol/l) and total testosterone (TT, nmol/l) during Phase 1 (treatment) and Phase 2 (recovery). Fixed effects include geographic cluster (Cluster A = reference), age group (>35 years = reference), time (weeks), and cluster × time interaction. Estimates are shown with standard errors (SE) and *F*-test *P*-values. Estimates ×10^−^^4^ are reported as in the SAS PROC MIXED output. Covariance structures: Toeplitz (heterogeneous autoregressive) for treatment phase; compound symmetry for recovery phase where applicable. SE: standard error. NS: not significant. Phase 1 here denotes the combined injection period of suppression and efficacy phase of the parent trial.

Regarding DHT, multilevel mixed-effects models confirmed a higher DHT level in the cluster-by-time interaction for Cluster B and a lower DHT level for Cluster C relative to Cluster A (Fig. 10).

**Figure 10.**
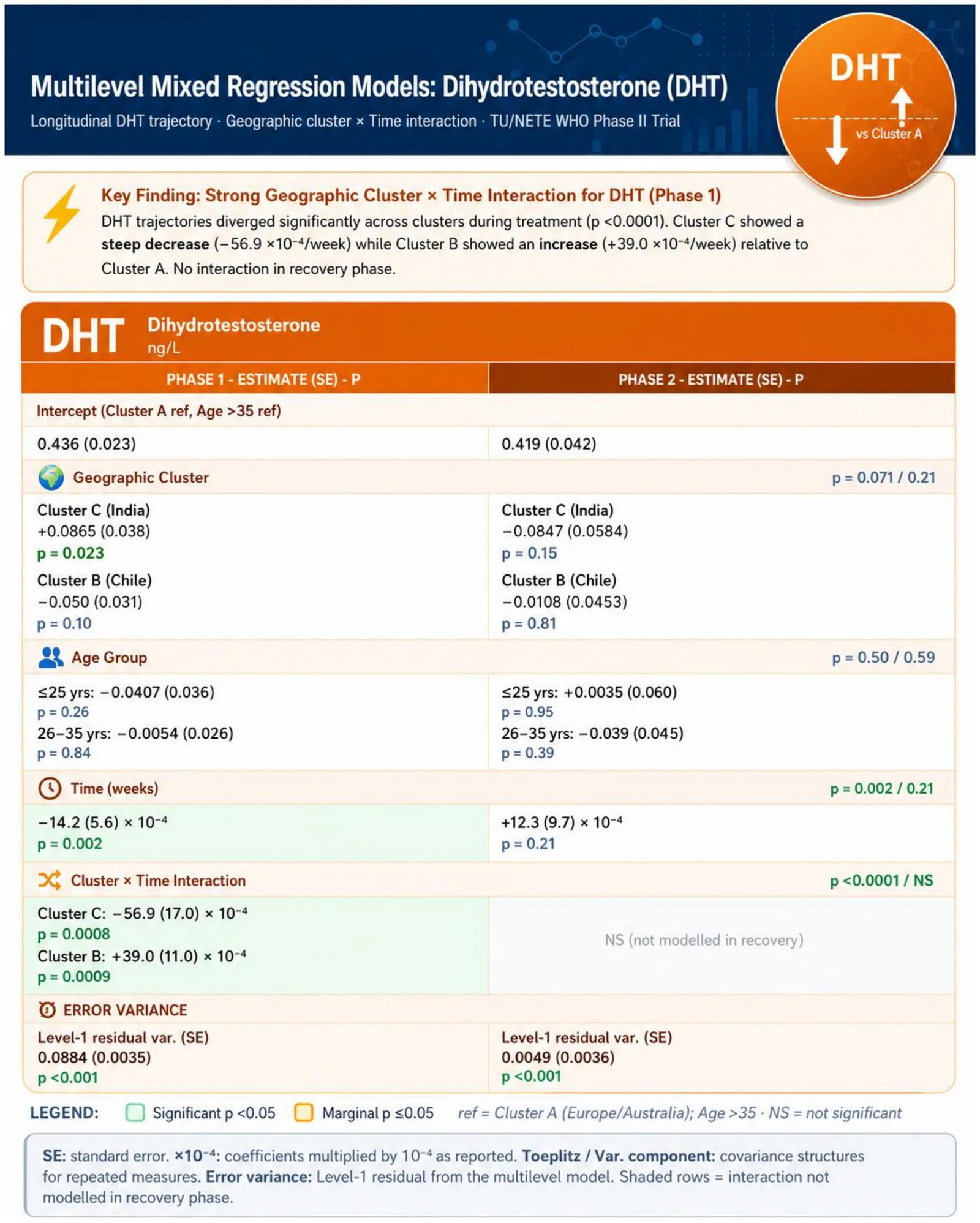
**Multilevel mixed-effects regression models for serum dihydrotestosterne**. Results of multilevel mixed-effects regression models for serum dihydrotestosterne (DHT) (ng/l) during Phase 1 (treatment) and Phase 2 (recovery). Fixed effects include geographic cluster (Cluster A = reference), age group (>35 years = reference), time (weeks), and cluster × time interaction. Estimates are shown with standard errors (SE) and *F*-test *P*-values. Estimates ×10^−^^4^ are reported as in the SAS PROC MIXED output. Covariance structures: Toeplitz (heterogeneous autoregressive) for treatment phase; compound symmetry for recovery phase where applicable. SE: standard error. NS: not significant. Phase 1 here denotes the combined injection period of suppression and efficacy phase of the parent trial.

The multilevel mixed-effects modelling of serum norethisterone concentrations (Fig. 11) confirmed the markedly higher levels in Cluster C compared to Cluster A (also see Fig. 8). Serum norethisterone concentrations differed by geographic cluster; concentrations in Clusters C were higher than in Clusters A and B (trend). Serum concentrations of estradiol did not differ significantly between Clusters (Fig. 11).

**Figure 11.**
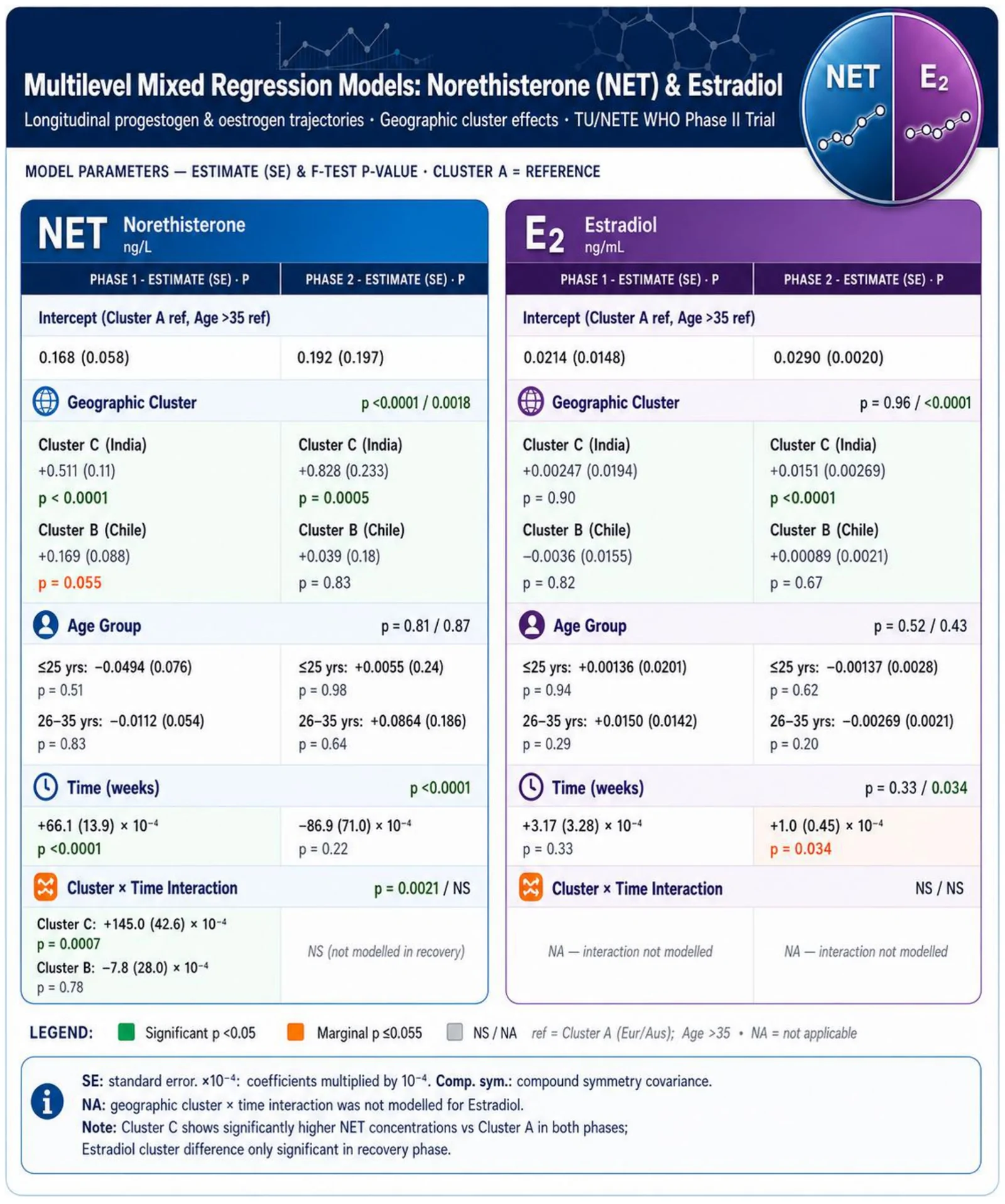
**Multilevel mixed-effects regression models for serum for serum norethisterone**. Results of multilevel mixed-effects regression models for serum for serum norethisterone (NET) and estradiol during Phase 1 (treatment) and Phase 2 (recovery). Fixed effects include geographic cluster (Cluster A = reference), age group (>35 years = reference), time (weeks), and cluster × time interaction. Estimates are shown with standard errors (SE) and *F*-test *P*-values. Estimates ×10^−^^4^ are reported as in the SAS PROC MIXED output. Covariance structures: Toeplitz (heterogeneous autoregressive) for treatment phase; compound symmetry for recovery phase where applicable. SE: standard error. NS: not significant. NA indicates that a given interaction was not applicable or not modelled for that analyte. Phase 1 here denotes the combined injection period of suppression and efficacy phase of the parent trial.

Figure 12 depicts the cumulative rate of first onset of increased libido stratified by geographic cluster/center, while Fig. 13 illustrates differences between different age groups as time-by-age interaction. These results are complemented by the multilevel mixed-effect models for libido increase presented in Fig. 14, which evaluates the independent and combined effects of key circulating hormones. Serum levels of measured testosterone and cFT as well as concentrations of DHT and estradiol as time-varying covariates contributed significantly to the observed increase in libido.

**Figure 12.**
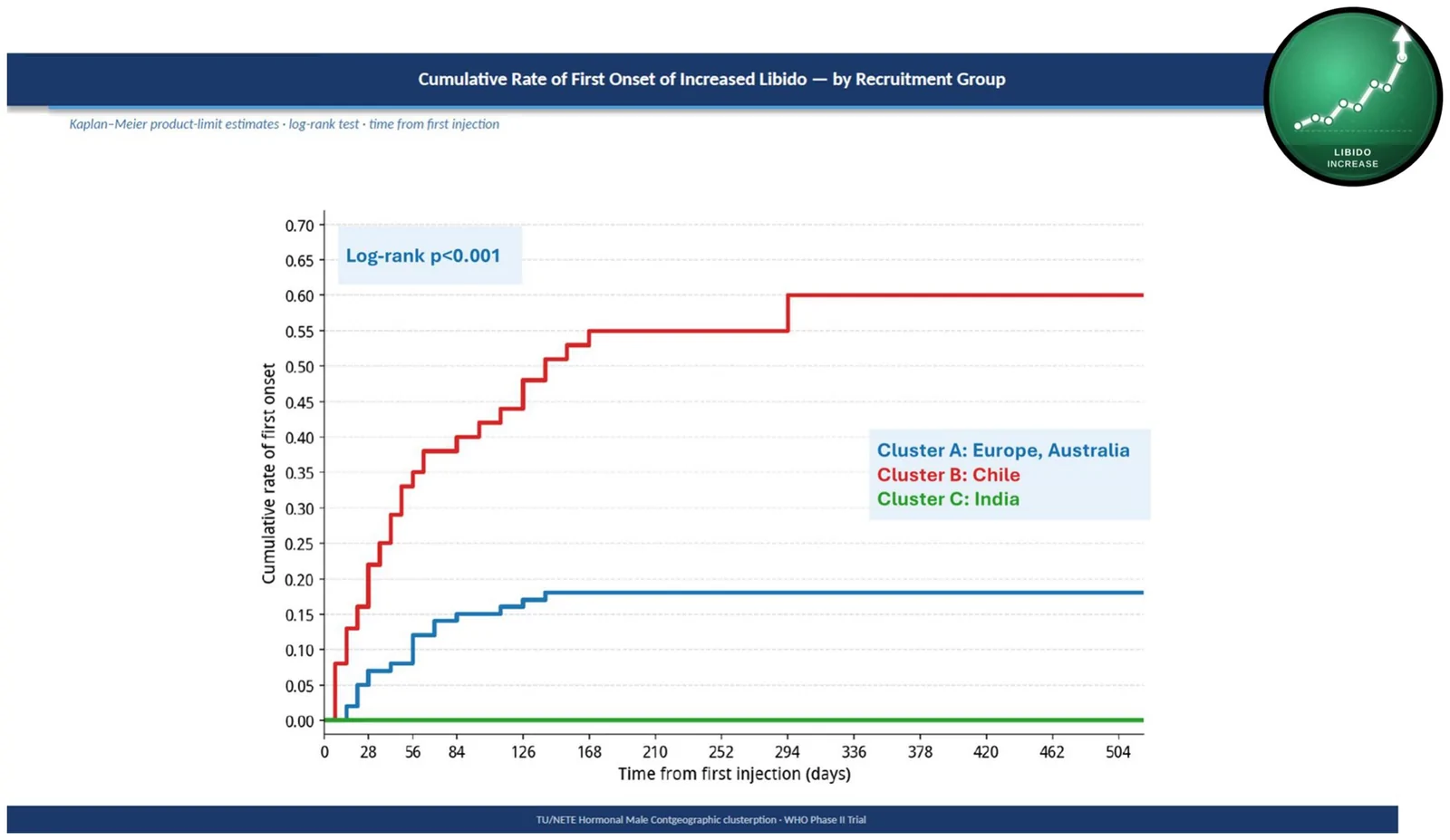
**Cumulative first onset of increased libido by recruitment group/geographic cluster**. Kaplan–Meier product-limit estimates for cumulative first onset of increased libido by recruitment group/geographic cluster. Cluster A comprised Europe/Australia, Cluster B Chile, and Cluster C India. Group differences were assessed by the log-rank test. As we are dealing with side effects independently appearing from Suppression and Efficacy, the models were calculated up to Day 504 to include subjects from India from where we had samples up to week 56 (Day 392) but reports on side effects until Day 504 (covering the depot effect of testosterone undecanoate).

**Figure 13.**
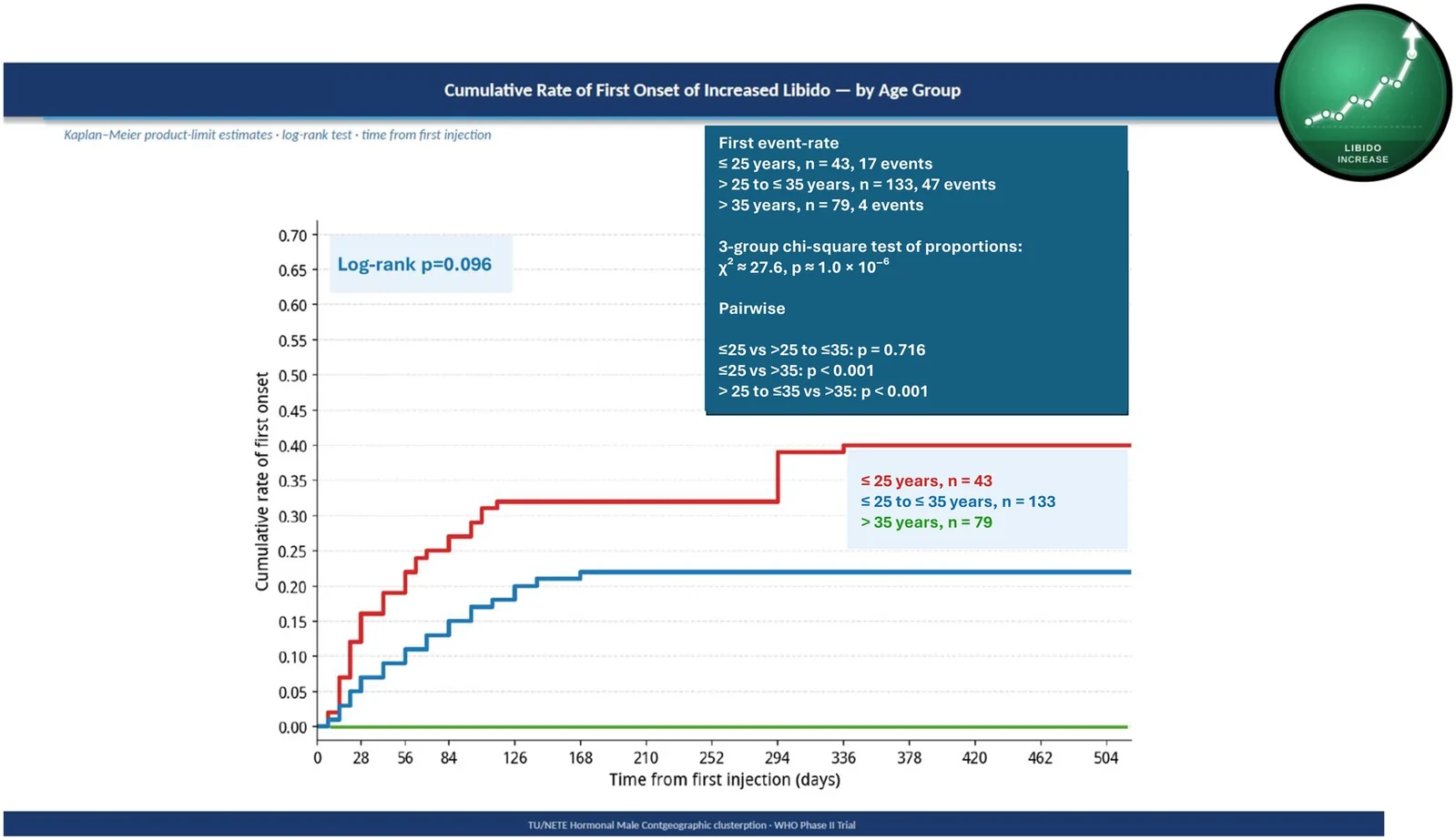
**Cumulative first onset of increased libido by age group**. Kaplan–Meier product-limit estimates for cumulative first onset of increased libido by age group (≤25 years, >25 to ≤35 years, and >35 years). The figure includes first event counts and selected pairwise comparisons. Group differences were assessed by the log-rank test. Note that these are first events only, as chi-square calculation would not have been possible with repeated events. A continuous model including all reported events, also repeated ones (as already reported in Legend of Fig. 2) is shown in Fig. 6c. As we are dealing with side effects independently appearing from suppression and efficacy, the models were calculated up to Day 504 to include subjects from India from where we had samples up to week 56 (Day 392) but reports on side effects until Day 504 (covering the depot effect of testosterone undecanoate).

**Figure 14.**
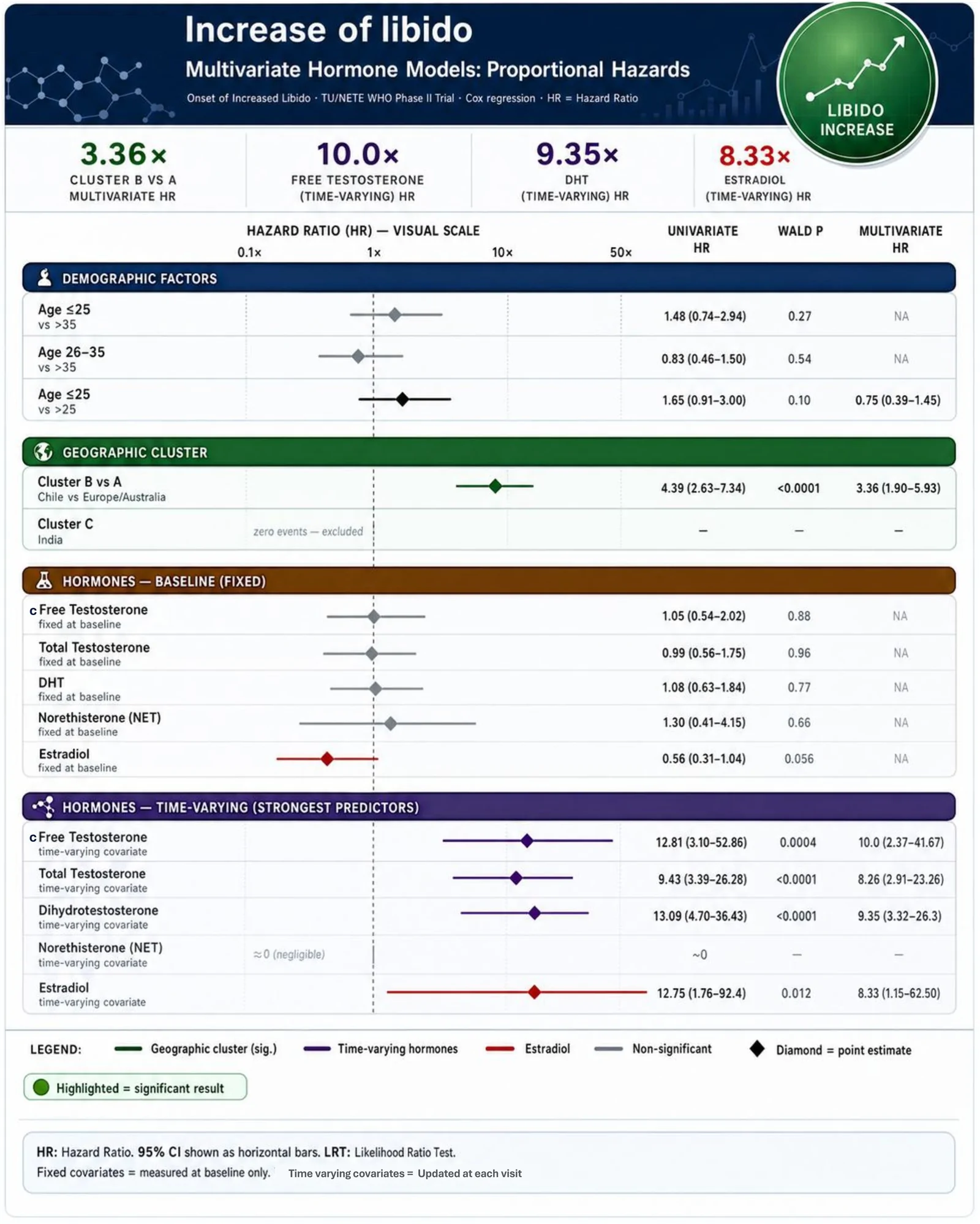
**Cox proportional hazards models for the report of increased libido**. Forest plot from time-varying covariate Cox proportional hazards models for the report of increased libido. Baseline (fixed) hormone levels and time-varying hormone covariates were evaluated alongside demographic and geographic factors. LRT denotes likelihood ratio test; NA, not applicable. HR denotes hazard ratio. Free testosterone represents calculated free testosterone according to Vermeulen (cFT).

The cumulative rate of first onset of decreased libido stratified by changes in serum DHT levels is illustrated in Fig. 15. A decreased serum concentration of DHT was defined as lower than 136 ng/l according to Wang *et al.* (2008) (reporting a male reference interval of 0.47–2.65 nmol/l for DHT by LC-MS/MS, which corresponds to 136–770 ng/l).

**Figure 15.**
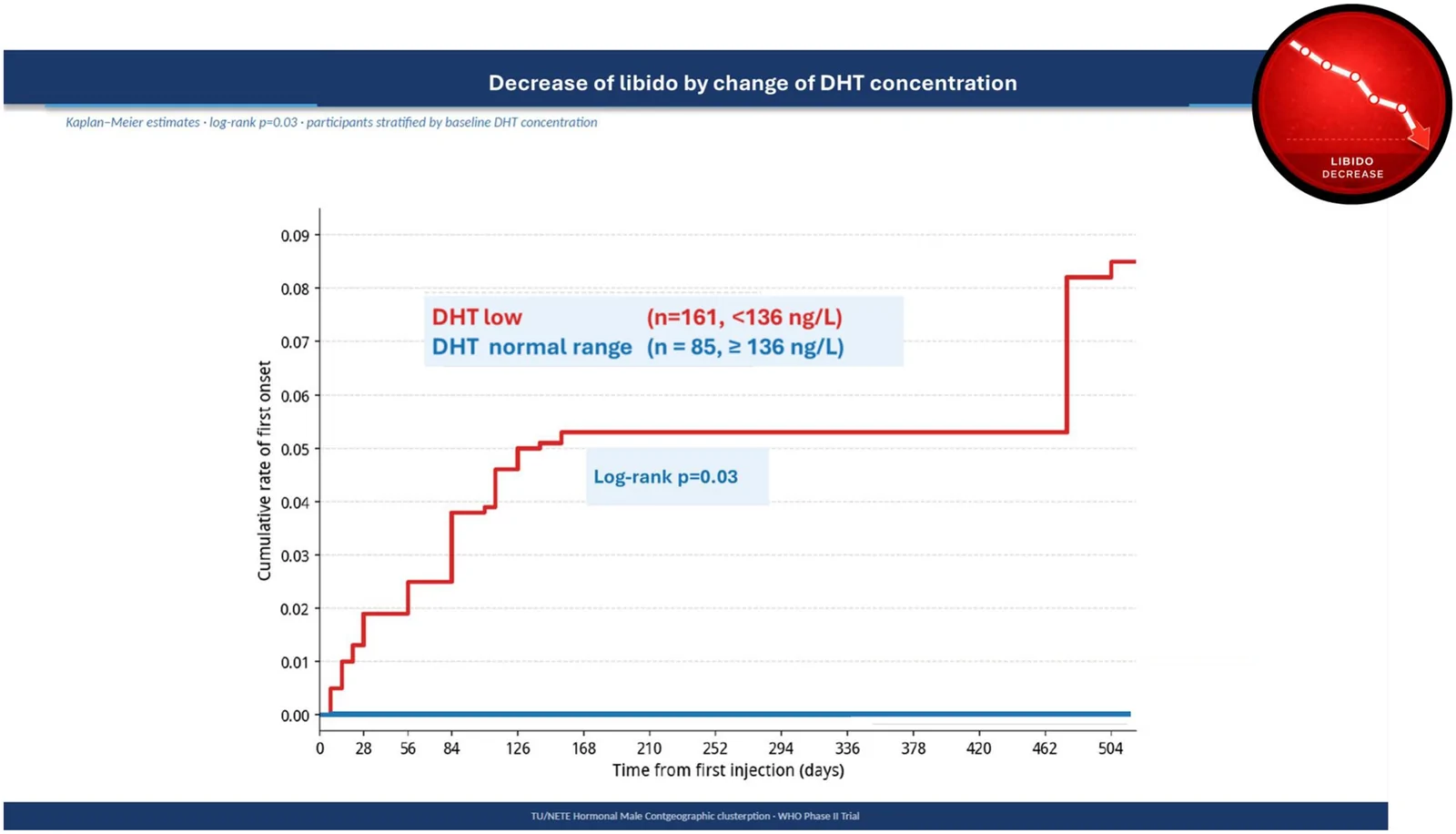
**Cumulative first onset of decreased libido stratified by change in serum DHT concentrations**. Kaplan–Meier estimates for cumulative first onset of decreased libido stratified by change in serum DHT concentrations. Participants were grouped as DHT low (<136 ng/l) or DHT normal range (≥136 ng/l). Threshold as previously described and determined by Wang *et al.* (2008). Group differences were assessed by the log-rank test. Potential non-linearity was examined using restricted cubic splines with K knots and a prespecified linear term because event numbers did not support spline modelling. The threshold of 136 ng/l corresponds to an external lower reference limit and was not developed as a diagnostic threshold for libido susceptibility. Its positive predictive value in this cohort was present, but most observations below the threshold were not followed by decreased libido. Accordingly, the dichotomized analysis is presented only as a descriptive sensitivity analysis and should not be used for individual prediction. Possible limitation: The number of decreased-libido events limits precise estimation of non-linear risk functions and precludes derivation of a stable clinical DHT cut-off. Any threshold suggested by these data would require validation in an independent prospective cohort. As we are dealing with side effects independently appearing from suppression and efficacy, the models were calculated up to Day 504 to include subjects from India from where we had samples up to week 56 (Day 392) but reports on side effects until Day 504 (covering the depot effect of testosterone undecanoate).

The cumulative incidence of mood disorders by geographic cluster is exhibited in Fig. 16 and multilevel mixed-effects models detail the results in Fig. 17.

**Figure 16.**
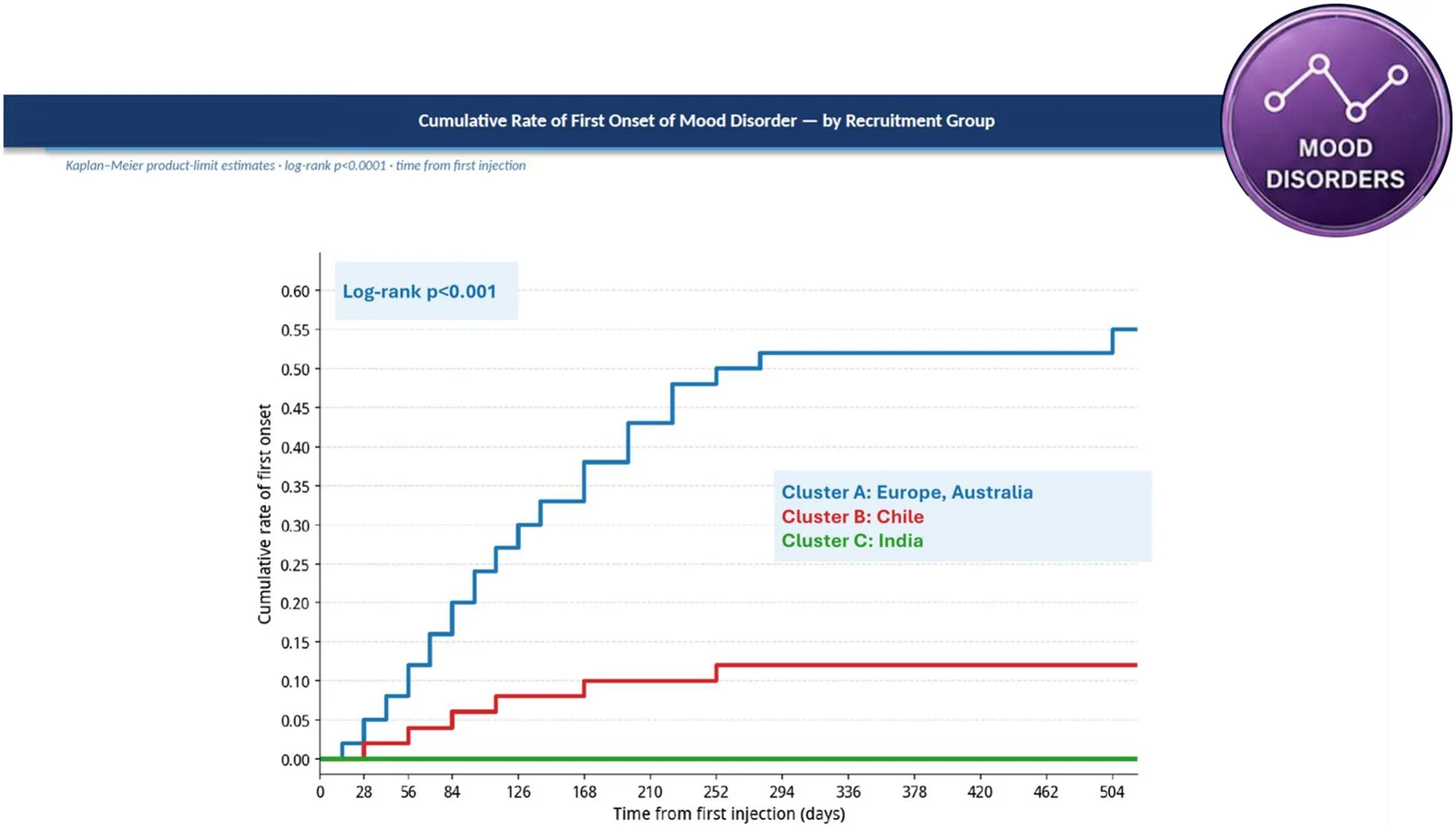
**Cumulative first onset of mood disorder by recruitment group/geographic cluster**. Kaplan–Meier product-limit estimates for cumulative first onset of mood disorder by recruitment group/geographic cluster. Cluster A comprised Europe/Australia, Cluster B Chile, and Cluster C India. Group differences were assessed by the log-rank test. Free testosterone represents calculated free testosterone according to Vermeulen (cFT). As we are dealing with side effects independently appearing from suppression and efficacy, the models were calculated up to Day 504 to include subjects from India from where we had samples up to week 56 (Day 392) but reports on side effects until Day 504 (covering the depot effect of testosterone undecanoate).

**Figure 17.**
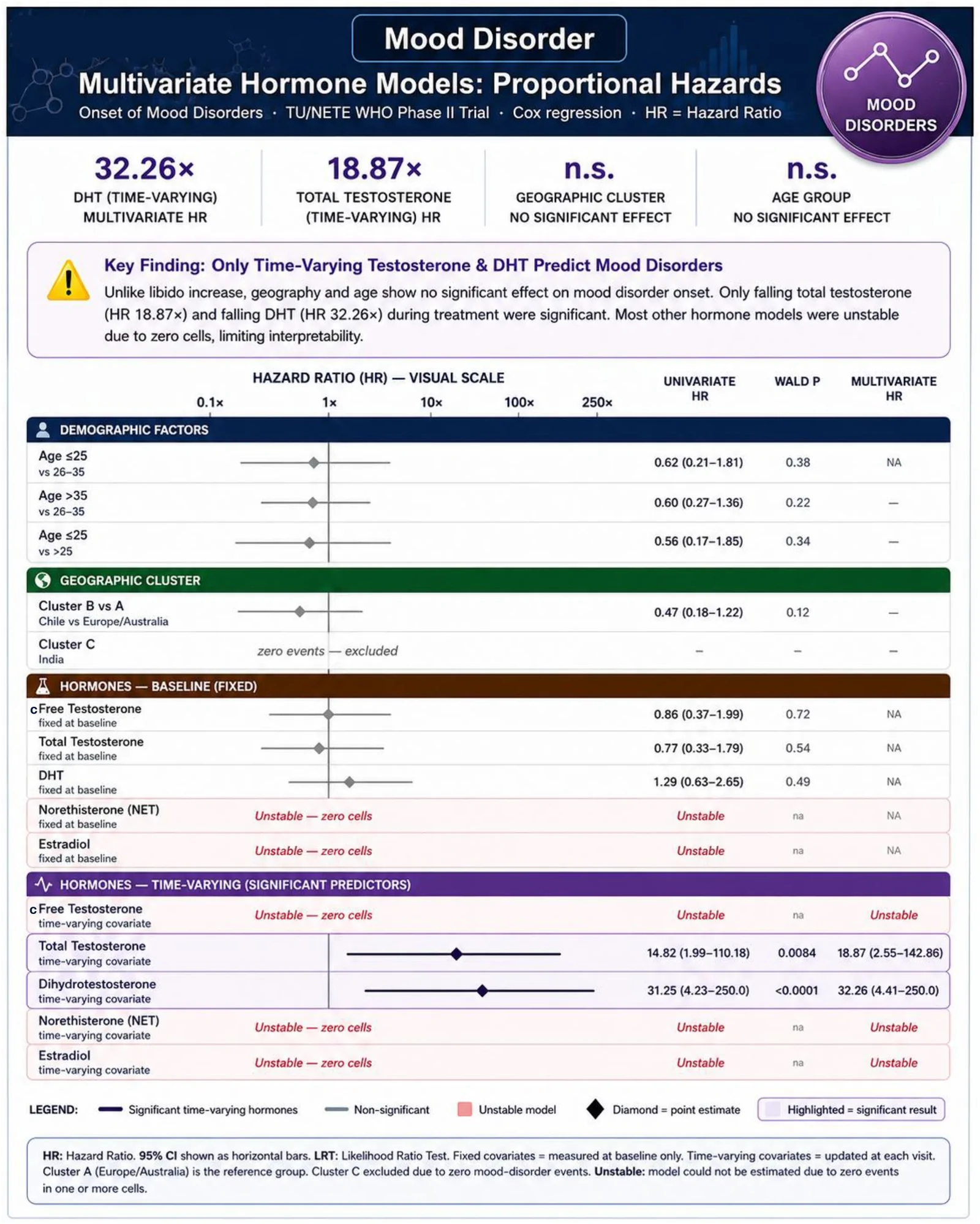
**Cox proportional hazards models for the first onset of mood disorder**. Forest plot from time-varying covariate Cox proportional hazards models for the first onset of mood disorder. Time-varying total testosterone (TT) and dihydrotestosterone (DHT) emerged as significant predictors in the displayed model; geographic cluster and age group were not significant. Unstable model indicates that the estimate could not be robustly computed because of sparse or zero-event cells: models for norethisterone (NET) and estradiol (E2). HR: hazard ratio. CI: confidence interval. LRT: likelihood ratio test. n.s.: not significant. Free testosterone represents calculated free testosterone according to Vermeulen (cFT).

A graphical summary of the results is presented in Fig. 18.

**Figure 18.**
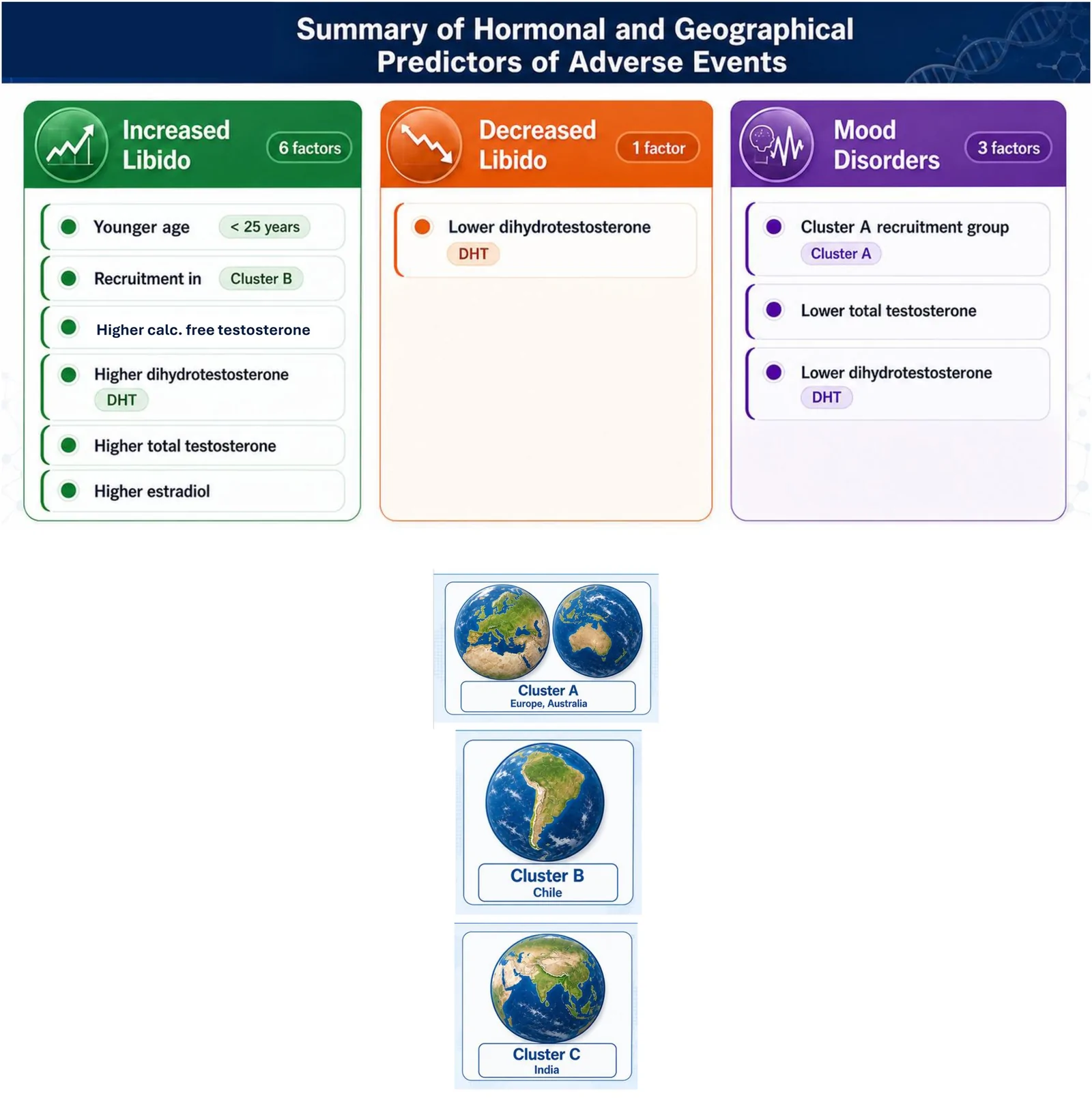
**Predictors of side effects**. Six significant predictors of increased libido could be described: younger age, recruitment in Cluster B, higher calculated free testosterone, higher dihydrotestosterone (DHT), higher total testosterone (TT), and higher estradiol. There was a single significant predictor of decreased libido (lower DHT), together with the predominant predictors of mood disorders (lower TT, lower DHT, Cluster A recruitment). The three geographic clusters are shown. Results are derived from the Kaplan–Meier and Cox proportional hazards analyses. Free testosterone represents calculated free testosterone according to Vermeulen (cFT).

## Discussion

In summary, GC-MS analysis of sex steroid concentrations and their associations with adverse effects, age, and geographic recruitment cluster yielded several important findings. Cluster B (South America) participants generally exhibited higher concentrations of cFT and DHT, along with a greater increase in libido, compared with those from Cluster A (Europe/Australia). Cluster C (India) participants demonstrated lower DHT concentrations and higher NET concentrations. Mood disorders were more prevalent among Cluster A participants and were associated with low DHT and cFT concentrations, elevated NET concentrations, and elevated estradiol concentrations. It bears emphasis, however, that the observed between-group differences do not necessarily reflect genetic or ethnic background alone: the geographically and culturally distinct nature of the recruitment cohorts means that environmental, dietary, and lifestyle factors may also have contributed.

Younger men (under 25 years) were more likely to experience an increase in libido. At other ages, the association between decreased libido and lower DHT concentrations is consistent with the established role of DHT in male sexual function (Corona and Maggi, 2022; Nguyen *et al.*, 2022). DHT is a potent androgen that plays a central role in the development and maintenance of male reproductive physiology, stimulating the growth of the male reproductive organs and the development of secondary sexual characteristics. DHT contributes to sexual desire and motivation by acting on androgen receptors in brain regions associated with sexual behaviour, thereby modulating sexual drive through both central and peripheral mechanisms (Sansone *et al.*, 2021; Corona and Maggi, 2022; Nguyen *et al.*, 2022). When DHT concentrations fall below the normal range, diminished sexual desire may result in reduced interest in sexual activity, decreased frequency of sexual ideation, and attenuated responses to sexual stimuli.

The observed association between mood disorders and lower DHT and measured testosterone concentrations in Cluster A participants, but not in those from Cluster C, suggests cluster-dependent variation in the relationship between androgens and mood regulation, though whether this reflects genetic background, geographic/environmental context, or both remains to be determined. The mechanisms underlying the influence of DHT on mood are complex and incompletely characterized; however, evidence indicates that sex hormones including DHT may exert modulatory effects on mood and emotional well-being (Ly *et al.*, 2001; Kunelius *et al.*, 2002; Zitzmann, 2020; Hauger *et al.*, 2022; Zito *et al.*, 2023) and may exert symptoms when lowered by medications, such as 5alpha-reductase inhibitors (DHT blockers) (Melcangi *et al.*, 2017; Pompili *et al.*, 2021). It is, nevertheless, also a widely held concept that endogenous circulating DHT is the overflow of local production of DHT in androgen-sensitive target tissue and not sufficient to have endocrine effects in its own right (Swerdloff *et al.*, 2017).

Plausible mechanisms include neuroactive properties of DHT, whereby it may interact with brain regions and neurotransmitter systems, particularly GABAergic pathways, involved in mood regulation (Frye and Walf, 2009; Wang, 2011; Melcangi *et al.*, 2017). Reduced DHT concentrations in susceptible Cluster A participants could disrupt such neuromodulatory processes and predispose to affective disturbance. These neuroactive properties may be particularly relevant in the context of the sharp, drug-induced decline in androgens experienced during TU/NETE treatment.

Genetic, geographic, or lifestyle-related differences in responsiveness to androgen fluctuations may account in part for these observations. For example, variation in the length of the androgen receptor CAG repeat, which modulates androgen receptor transactivation capacity and has been linked to mood vulnerability (Zitzmann, 2020). The relative absence of mood disturbances in Cluster C could reflect genetic variants conferring differential sensitivity to DHT-mediated neuromodulation or alternatively to geographic, dietary, or cultural factors, including differences in macronutrient and phytoestrogen intake, habitual physical activity, altitude, or cultural attitudes towards health reporting. Cross-cultural variation in testosterone concentrations between populations has been documented and attributed in part to energy availability and lifestyle factors (Alvarado, 2013).

It can be speculated that the associations of mood disorders with lower estradiol and higher NET concentrations further suggest that mood disorders in Cluster A may reflect a multifactorial hormonal perturbation. Given the known interactions among sex hormones, concurrent imbalances in multiple hormones may act synergistically to predispose to affective disturbances. It must be emphasized that mood disorders are multifactorial in aetiology, arising from a complex interplay of genetic, hormonal, environmental, and psychological determinants. While the present findings suggest potential associations between DHT, estradiol, NET, and mood disorders in Cluster A, further research is required to elucidate the underlying mechanisms and establish causal relationships.

The present investigation has generated important insights into the potential for individualized medicine in hormonal male contraception. The observed adverse effects and the consequent premature termination of the trial underscore the complexity of hormonal modulation and the imperative to tailor contraceptive approaches to individual physiological variation. A central finding was the marked variability in adverse effects across clusters: Cluster A exhibited mood disorders at the highest frequency, Cluster B reported the greatest increase in libido, and Cluster C the lowest libido. Concomitant differences in hormone concentrations, including lower DHT and higher NET in Cluster C, were also documented.

The most actionable observation is that not a single baseline androgen concentration, but the dynamic on-treatment trajectory of testosterone and dihydrotestosterone influences side effects.

Of particular relevance is the role of norethisterone: in addition to its progestogenic actions, NET has been recognized as a 5α-reductase inhibitor, thereby reducing the conversion of testosterone to the more potent androgen DHT (Chao and Page, 2016; Bania *et al.*, 2025). This 5α-reductase inhibitory activity, also exploited therapeutically in male pattern baldness and benign prostatic hyperplasia, may contribute to the DHT suppression and associated adverse effects observed in the present study.

The most clinically actionable observation in this secondary analysis was that treatment-emergent androgen trajectories, rather than baseline hormone concentrations alone, were associated with sexual and mood-related outcomes. This distinction reframes susceptibility as a potentially monitorable process: a participant may enter treatment with an unremarkable baseline profile yet develop an adverse clinical response as testosterone or DHT falls during therapy. Future male contraceptive trials should therefore evaluate prespecified on-treatment androgen trajectories, together with symptoms, rather than relying exclusively on baseline screening thresholds. The recruitment centre remained associated with heterogeneity in hormonal profiles and selected outcomes, but should be interpreted as a composite contextual variable rather than as an intrinsically causal determinant.

Possible limitations of our analyses include: their *post hoc*, secondary nature; restriction to participants with available centrally stored serum samples; and the possibility that geographic centre captures unmeasured genetic, nutritional, environmental, cultural, behavioural, and reporting-related influences. Accordingly, the findings should be interpreted as hypothesis-generating associations rather than causal effects. Nevertheless, the repeated association of dynamic DHT exposure with sexual and mood-related outcomes across complementary longitudinal and time-to-event models provides a coherent biological signal that warrants prospective awareness in male contraception.

‘Recruitment centre’ should be understood as a composite contextual variable rather than an isolated causal exposure. The study centres differed in latitude, hemisphere, photoperiod, climate, diet, lifestyle, and cultural contexts, while Cluster A combined European and Australian sites. The present analysis therefore cannot attribute between-centre hormonal differences specifically to geography, ethnicity season, or any single environmental factor.

Age as a modifier of events requires stratification, while preserving the central result that dynamic androgen changes were associated with outcomes after accounting for measured age.

## Conclusion

These findings carry significant implications for the development of individualized approaches to male contraception. Understanding the differential responses across geographically and culturally distinct populations can inform the development of population-context-specific regimens, potentially through adjustment of hormone doses or combinations to minimize adverse effects while maximizing effectiveness. Personalized medicine in male contraception may involve prospective assessment of an individual’s baseline hormone concentrations, genetic predispositions, and hormonal response profiles to guide the selection of the most appropriate regimen (Zitzmann, 2020; Hauger *et al.*, 2022). Such tailored approaches could optimize both safety and efficacy.

## Data Availability

The data underlying this article will be shared on reasonable request communicated to World Health Organization (WHO).
